# Knowledge, Attitudes, and Practices Regarding Gut Microbiota and Probiotics Among Ecuadorian Medical Students

**DOI:** 10.3390/healthcare14111551

**Published:** 2026-06-02

**Authors:** María Nicole Solis, Miroslava Anna Šefcová, César Marcelo Larrea-Álvarez, Renáta Szaboová, Róbert Herich, Marco Larrea-Álvarez

**Affiliations:** 1 Facultad de Ciencias de la Salud, Carrera de Medicina, Universidad Espíritu Santo, Samborondón 092301, Ecuador; 2 Department of Materials Science, E.T.S.I. Caminos, Universidad Politécnica de Madrid, C/Profesor Aranguren 3, 28040 Madrid, Spain; 3 Department of Biology and Physiology, University of Veterinary Medicine and Pharmacy in Košice, 041 81 Košice, Slovakia; 4 Department of Morphological Disciplines, University of Veterinary Medicine and Pharmacy in Košice, 041 81 Košice, Slovakia

**Keywords:** probiotics, gut microbiota, KAP, medical students, cross-sectional study, Ecuador

## Abstract

**Background/Objectives**: The gut microbiota is fundamental to human health. Probiotics are key interventions for modulating microbial balance, requiring future healthcare professionals to understand these concepts. Evidence evaluating the knowledge, attitudes, and practices (KAP) of medical students in Ecuador is limited. This study assessed these aspects among students in Greater Guayaquil. **Methods**: A cross-sectional study was conducted at a university in Samborondón between August and November 2025. A previously published questionnaire was used to collect data on microbiota and probiotic knowledge, attitudes, and personal practices. Data were analyzed using descriptive statistics and multivariable regression. **Results**: Among 382 participants, the mean knowledge score was 22.76 ± 9.1 out of 44 (52.7%). Higher scores were associated with advanced academic standing and prior formal instruction. Significant gaps were observed in probiotic safety and microbiota–drug interactions. Attitudes were positive (median 10/16), with strong support for enhanced training. Probiotic use was common, though uncertainty about product composition and administration persisted. **Conclusions**: Medical students possess moderate yet uneven knowledge of microbiota and probiotics, alongside positive attitudes. These findings suggest the need for greater emphasis on microbiota science, probiotic safety, and drug interactions within medical curricula.

## 1. Introduction

The microbiota refers to the community of microorganisms that colonizes a specific site in the host, whose composition is influenced by factors such as genetics, age, lifestyle, diet, antibiotic use, and mode of delivery at birth [1,2]. The gut microbiota exhibits considerable diversity across individuals and along the gastrointestinal tract and plays key roles in host physiology, including nutrient digestion, intestinal barrier maintenance, immunity, neurological signaling, and drug metabolism [1,3]. Bacteria are predominant, particularly members of the Firmicutes, Bacteroidetes, Actinobacteria, and Proteobacteria phyla [2,4]. Host health is critically dependent on the composition and functional potential of the gut microbiota. When this microbial community becomes imbalanced, a phenomenon known as dysbiosis, it may initiate or exacerbate pathological processes [1,5]. Dysbiosis is an established contributory factor in metabolic and neoplastic diseases, including obesity, diabetes, and colorectal cancer [6,7]. Among strategies to restore the gut microbiota, probiotics have been proposed as a promising, non-invasive option [8].

Probiotics are defined by the Food and Agriculture Organization (FAO) and the World Health Organization (WHO) as “live microorganisms that, when administered in adequate amounts, confer a health benefit on the host” [9], with later refinements emphasizing well-defined strains and sufficient viable counts [10]. While bifidobacteria and lactic acid bacteria are most commonly used, other genera such as *Enterococcus*, *Lactococcus*, *Streptococcus*, *Propionibacterium*, and the yeast *Saccharomyces* are also utilized [11]. Probiotics may be combined with prebiotic fibers (synbiotics) to enhance gut health [12,13]. Their beneficial effects include strengthening the intestinal barrier, stabilizing the microbiota, inhibiting pathogens, and modulating immune responses [14,15,16,17,18,19]. Probiotics have shown therapeutic potential in gastrointestinal disorders such as diarrhea and inflammatory bowel disease (IBD) [20,21], as well as in extra-intestinal conditions including allergies and vaginal infections [22,23]. They may also influence pharmacokinetics by affecting drug absorption and metabolism [24,25], although their effects remain strain-specific and dose-dependent [8,26].

Previous studies among university and healthcare students generally report moderate knowledge of gut microbiota and probiotics, with relatively few individuals achieving high proficiency [27,28,29,30,31]. Knowledge tends to improve with academic progression and microbiology training [28,29,32,33], yet important gaps persist, particularly regarding specific indications, safety considerations, and microbiota–drug interactions [27,28,29,31,32,33]. Attitudes toward probiotics and microbiota-related education are generally positive [28,31,34,35,36,37], although misconceptions remain among individuals with limited knowledge [27,38]. Despite this, many students report insufficient formal training, and favorable perceptions often coexist with only moderate scientific understanding [28,35,36,39,40]. Healthcare students frequently report personal use of probiotic products, indicating active engagement with these interventions [27,41,42]. Such use occurs even among those with moderate knowledge, suggesting that behavioral adoption may precede full scientific understanding [27,41]. However, inconsistencies in probiotic-related practices are common, including limited awareness of specific products, administration timing, and interactions with medications [29]. Probiotic use is influenced by knowledge, health consciousness, and access to information [37,43], with information often obtained from media or informal sources rather than healthcare professionals [29,44,45,46,47,48]. However, evidence on these aspects remains limited in specific regional contexts, particularly in Latin America, where data on medical students are scarce.

Ecuador’s higher education system includes public and private universities offering undergraduate and postgraduate programs. Medical training follows standardized competencies and typically involves five to six years of integrated theoretical and clinical education, culminating in a supervised internship year [49]. Studies among Ecuadorian university students on topics such as antibiotic awareness [50,51], genetics [40,52], COVID-19 knowledge [53], and analgesic self-medication [54] consistently report moderate knowledge levels with persistent misconceptions, highlighting the need for targeted educational interventions.

Despite increasing global interest in the gut microbiota and probiotics, studies assessing knowledge, attitudes, and practices among medical students in Ecuador remain limited. To our knowledge, only one study has evaluated probiotic-related knowledge among dental students in Ambato, reporting notable gaps [55]. Although probiotic products are widely accessible in Guayaquil, research in this area remains scarce [56]. Given these gaps, there is a need to better characterize knowledge, attitudes, and practices regarding gut microbiota and probiotics among medical students in this setting. We hypothesized that medical students would demonstrate variable knowledge of gut microbiota and probiotics, and that knowledge levels would differ according to academic training and prior exposure to formal instruction. We further hypothesized that discrepancies would exist between knowledge and reported practices. Accordingly, this study assessed knowledge, attitudes, and practices (KAP) regarding these topics among medical students in Samborondón, Greater Guayaquil, using a previously published questionnaire [29].

## 2. Materials and Methods

### 2.1. Ethical Approval and Informed Consent

The methodology of the present study was approved by the Human Research Ethics Committee of UEES (CEISH UEES) (Reference code: C-UEES-25-09). Participation was voluntary, and informed consent was obtained electronically from all participants prior to accessing the survey. Participants were informed about the purpose of the study, the anonymous nature of the questionnaire, and their right to withdraw at any time without consequence. The study complied with international ethical standards for human subjects research outlined in the Declaration of Helsinki.

### 2.2. Study Instrument

The present study was carried out using a previously published instrument designed to assess the knowledge, attitudes, and practices of medical students regarding gut microbiota, probiotics, and their interaction with drugs [29], which had been developed based on prior surveys and pretested in the original study to assess content, readability, comprehension, and questionnaire design [29]. The questionnaire (Supplementary Materials, Survey) consisted of four sections. Section (1) demographics, including gender, year of the study (1st–6th years), as well as general information about lifestyle, the presence of chronic diseases, self-assessment of knowledge on probiotics and microbiota (very good, good, fair, poor, and very poor), and sources of information about probiotics. Section (2) evaluated knowledge using multiple-choice questions and true/false (T/F) items. The multiple-choice questions addressed definitions of gut microbiota, prebiotics, synbiotics, and probiotics, as well as the safety of probiotics. Additional items covered topics related to the composition of the gut microbiota, bacterial species used as probiotics and their efficacy in certain conditions, and their interactions with drugs. A single point was assigned to each correct response, while incorrect answers and those marked as “do not know” received 0 points. The maximum possible score for this section was 44 points. Section (3) assessed attitudes toward the use of probiotics using a Likert scale, and Section (4) addressed prior experience with probiotics. For the attitudes section, four points were assigned for the “strongly agree” option, while 3, 2, and 1 points were assigned to the “agree,” “disagree,” and “strongly disagree” options, respectively. In accordance with Bloom’s classification system, scores were categorized as good (≥80%), moderate (60–79%), or poor (<60%) [57]. The maximum score for this section was 16 points. The instrument underwent pilot testing with a cohort of 20 students to evaluate the comprehensibility of its Spanish translation and estimate the time required for completion. Input from this phase prompted slight wording refinements, although the underlying item content and scoring methodology remained unchanged.

### 2.3. Setting, Participants, and Procedure

This study was conducted from August to November 2025 in Samborondón, Greater Guayaquil, Ecuador, among Medicine students from one university. The identity of the institution has been kept confidential to safeguard participant privacy, moderate potential bias, and enhance the broader applicability of the results. At the time of the study, the School of Medicine had a total enrollment of 1361 students, all of whom were eligible and invited to participate through official university communication channels (institutional email and academic platforms). A total of 382 valid responses were obtained, corresponding to a response rate of 28.1%. The required sample size was estimated at 300 using the Raosoft (2004) (https://raosoftcalculator.com) [58] online sample size calculator, assuming a 50% response distribution, a 95% confidence level, and a 5% margin of error; the achieved sample exceeded this threshold. Given the exploratory aim of this research, a non-probability convenience sampling approach was employed. Participation was voluntary, which may have introduced self-selection bias, as students with prior interest in microbiota or probiotics could have been more likely to respond. The questionnaire was administered in Spanish and distributed using the Google Forms platform.

### 2.4. Statistical Analysis

Frequencies and percentages were used to summarize categorical variables, and differences between groups were assessed using chi-square tests. Comparisons were conducted across predefined demographic and educational groups, including sex (female vs. male), academic level (basic, intermediate, advanced), prior instruction (none, limited, extensive), and self-assessed knowledge categories, to assess differences in knowledge and attitude scores and to examine variables associated with these outcomes. Homogeneity of variance and normality were evaluated using Levene’s test and the Shapiro–Wilk test, respectively. Knowledge score data were normally distributed and demonstrated homogeneity of variance; therefore, differences between groups were assessed using one-way analysis of variance (ANOVA) followed by Tukey’s post hoc test. These results are reported as means ± standard deviation (SD). In contrast, attitude score data were not normally distributed but met the assumption of homogeneity of variance. Consequently, differences between groups were evaluated using the Kruskal–Wallis test followed by Wilcoxon rank-sum tests for pairwise comparisons. These results are reported as medians with interquartile ranges (IQR). Effect sizes with corresponding confidence intervals were calculated to quantify variation among demographic groups. The η^2^ values of 0.01, 0.06, and 0.14 were interpreted as small, moderate, and large effects, respectively [59,60]. Predictors of knowledge and attitude scores were examined using stepwise multiple linear regression (MLR). Variables showing associations in preliminary analyses (*p* ≤ 0.05) were entered into the regression models using a stepwise selection procedure based on statistical criteria. Separate models were constructed for knowledge and attitude scores as dependent variables. Prior to model fitting, the standard assumptions of multiple linear regression, including linearity, normality of residuals, and homoscedasticity, were evaluated. Pearson’s correlation coefficient (r) was used to assess the strength of the association between knowledge and attitude scores. Statistical significance was set at *p* ≤ 0.05. Data processing and statistical analyses were performed using MATLAB^®^ version 9.9.9341360 (MathWorks, Natick, MA, USA). Graphical visualizations were generated using Matplotlib version 3.0.3 within the Python programming environment (Python Software Foundation, Fredericksburg, VA, USA).

## 3. Results

### 3.1. Demographics and Sources of Information

A total of 382 surveys were completed (Table 1) (Supplementary Results, Raw data). The sample included 63% female participants and was distributed across academic years as follows: first–second (57%), third–fourth (30%), and fifth–sixth (13%). While the distribution of sex across academic levels was not a focus of the present study, participants of both sexes were included across all years of training. Regarding lifestyle, 51% rated their habits as Fair, 28% as Good, approximately 10% as Poor or Very Poor, and a similar proportion as Very Good. Chronic diseases were reported by 12% of participants. Prior instruction on probiotics or microbiota was reported as none (48%), limited (32%), or extensive (20%). Self-assessed knowledge was rated as Fair (37%), Poor (30%), Good (19%), Very Poor (10%), and Very Good (4%). Reported sources of information included the university (27.9%, *n* = 217), internet (25.5%, *n* = 198), physicians (17.9%, *n* = 139), scientific articles (15.4%, *n* = 120), and friends or family (9.5%, *n* = 74); pharmacists accounted for 3.6% (*n* = 28), and conferences and TikTok 0.1% each (*n* = 1).

### 3.2. Knowledge of Medical Students Regarding Gut Microbiota and Probiotics

The overall mean knowledge score was 22.76 (SD = 9.1) out of 44 points, corresponding to 52.7% of the maximum score. No significant differences were observed by sex (mean difference: 1.65; 95% CI: −0.23 to 3.53), lifestyle habits, or chronic disease status. Academic level was associated with knowledge scores, with students in their 5th and 6th years scoring approximately six points higher than those in the basic years. Prior exposure to lectures on microbiota and probiotics was also associated with higher scores, with students reporting extensive instruction scoring approximately five points higher than those with limited or no instruction. Self-assessed knowledge showed a similar pattern, with higher scores among students reporting Good knowledge compared to those reporting Poor or Very Poor knowledge; no significant differences were observed for those reporting Very Good knowledge (Table 1). These associations remained significant in multivariable linear regression analysis (Table 2). After adjustment, advanced academic level and extensive prior instruction were associated with higher scores, whereas lower self-rated knowledge (Poor or Very Poor) was associated with lower scores. The model explained 16.9% of the variance (adjusted R^2^ = 0.151).

**Figure 1:**
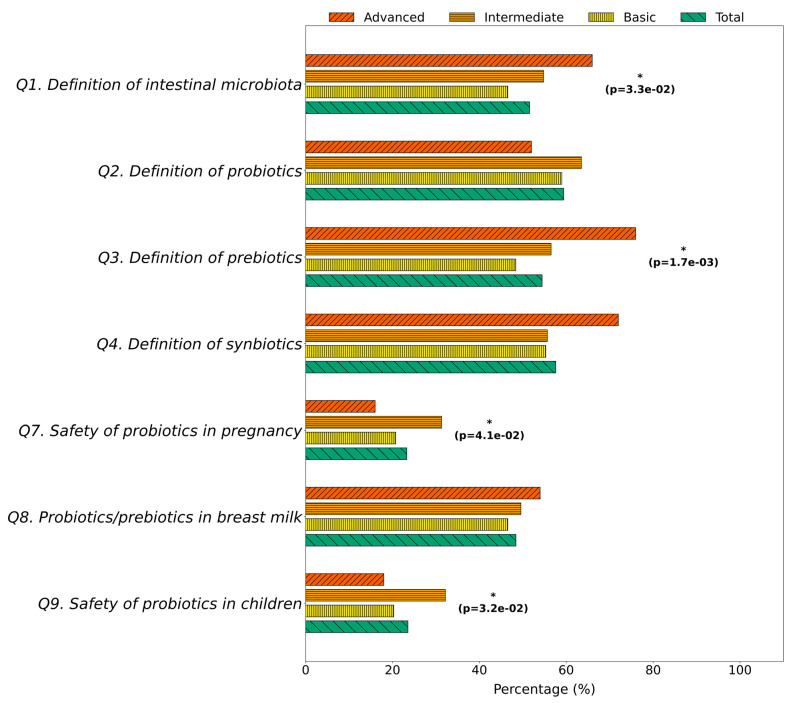
Students’ correct response rate on basic and applied probiotics questions. * indicates significant differences between academic levels.

Correct response rates across items ranged from 23.3% to 59.4% (Figure 1). The highest proportions of correct responses were observed for the definitions of probiotics (Q2, 59.4%) and synbiotics (Q4, 57.6%), whereas lower proportions were observed for safety-related items, including probiotics in pregnancy (Q7, 23.3%) and in children (Q9, 23.6%). Differences by academic level were identified for prebiotics (Q3, *p* = 0.002) and gut microbiota (Q1, *p* = 0.03), with higher proportions of correct responses among advanced students. Differences were also observed for safety-related items (Q7, *p* = 0.04; Q9, *p* = 0.03). No differences were found for probiotics (Q2), synbiotics (Q4), or breast milk content (Q8). When stratified by self-assessed knowledge, correct response rates increased across categories for several items (Supplementary Figure S1); however, for safety-related items, correct responses among students reporting Good or Very Good knowledge remained below 45%.

**Figure 2:**
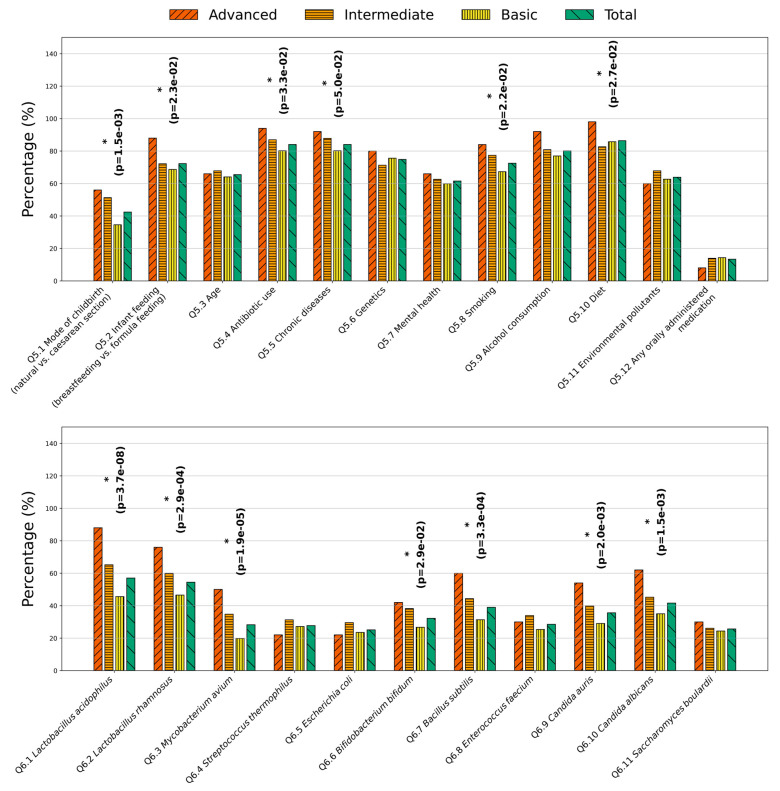
Knowledge assessment on microbiota and probiotics knowledge, including factors affecting gut microbiota composition (**upper panel**) and microorganisms recognized as probiotics (**lower panel**). * indicates significant differences between academic levels.

The percentage of students who correctly identified factors affecting gut microbiota composition varied widely, from 13.4% to 86.4%. (Figure 2, upper panel). The highest proportions were observed for diet (Q5.10, 86.4%), antibiotic use (Q5.4, 84.0%), and chronic diseases (Q5.5, 84.0%), while lower proportions were observed for orally administered medications (Q5.12, 13.4%) and mode of childbirth (Q5.1, 42.4%). Differences by academic level were identified for diet (Q5.10, *p* = 0.027), antibiotic use (Q5.4, *p* = 0.033), chronic diseases (Q5.5, *p* = 0.050), smoking (Q5.8, *p* = 0.022), infant feeding (Q5.2, *p* = 0.023), and mode of childbirth (Q5.1, *p* = 0.002), with higher proportions of correct responses among advanced students. When stratified by self-assessed knowledge, correct response rates increased across categories for several items (Supplementary Figures, Figure S2). Among students reporting Good or Very Good knowledge, correct responses were 40.0% for mode of childbirth (Q5.1) and 6.7% for orally administered medications (Q5.12). Recognition of microorganisms considered probiotics is presented in Figure 2 (lower panel). Correct response rates were highest for *Lactobacillus acidophilus* (Q6.1, 57.1%) and *Lactobacillus rhamnosus* (Q6.2, 54.5%), and lowest for *Saccharomyces boulardii* (Q6.11, 25.7%) and *Escherichia coli* (Q6.5, 25.1%). Differences by academic level were observed for *L. acidophilus* (Q6.1, *p* < 0.001), *L. rhamnosus* (Q6.2, *p* < 0.001), *Candida albicans* (Q6.10, *p* = 0.002), *Bacillus subtilis* (Q6.7, *p* < 0.001), *Candida auris* (Q6.9, *p* = 0.002), *Bifidobacterium bifidum* (Q6.6, *p* = 0.029), and *Mycobacterium avium* (Q6.3, *p* < 0.001), with higher proportions of correct responses among advanced and intermediate students. No differences were observed for *Enterococcus faecium* (Q6.8), *Streptococcus thermophilus* (Q6.4), *S. boulardii* (Q6.11), or *E. coli* (Q6.5). Stratification by self-assessed knowledge showed higher correct response rates among students reporting greater knowledge (Supplementary Figures, Figure S2). Correct identification of *S. boulardii* remained below 40% among students reporting Good or Very Good knowledge, and *E. coli* did not exceed 31% across categories.

**Figure 3:**
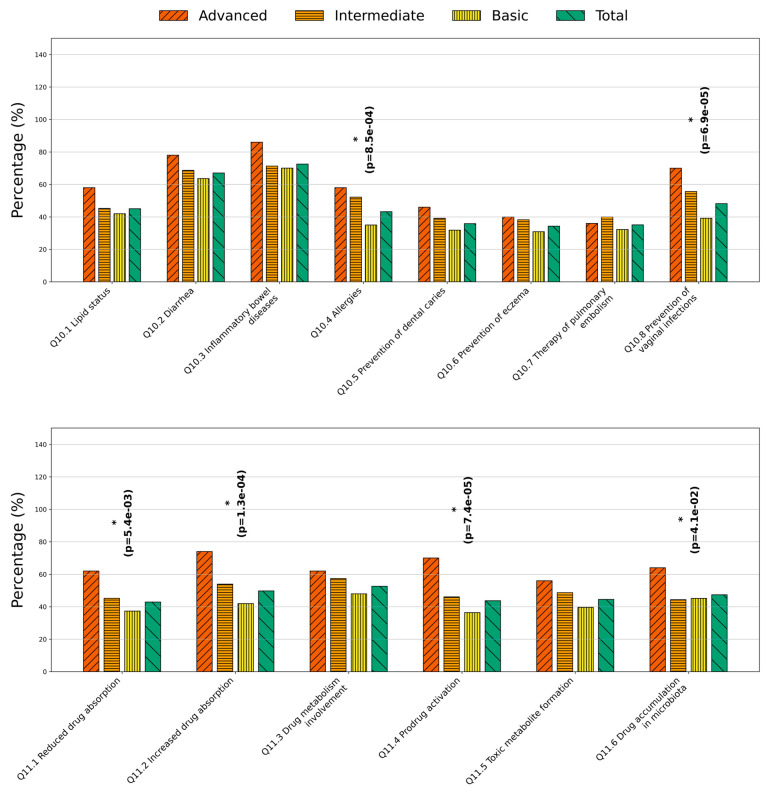
Accuracy on statements regarding probiotics in clinical practice with the upper panel showing therapeutic efficacy and the lower panel showing pharmacological interactions. * indicates significant differences between academic levels.

Knowledge of probiotic efficacy across clinical indications ranged from 34.3% to 72.5% (Figure 3, upper panel). The highest proportions were observed for inflammatory bowel diseases (Q10.3, 72.5%) and diarrhea (Q10.2, 67.0%), and lower proportions for prevention of eczema (Q10.6, 34.3%) and pulmonary embolism (Q10.7, 35.1%). Differences by academic level were identified for prevention of vaginal infections (Q10.8, *p* = 0.0001) and allergies (Q10.4, *p* = 0.0009), with higher proportions of correct responses among advanced students. No differences were observed for inflammatory bowel diseases (Q10.3), diarrhea (Q10.2), lipid status (Q10.1), prevention of dental caries (Q10.5), pulmonary embolism (Q10.7), or prevention of eczema (Q10.6). When stratified by self-assessed knowledge, correct response rates increased across categories for several indications (Supplementary Figures, Figure S3). Among students reporting Good or Very Good knowledge, correct responses did not exceed two-thirds for prevention of eczema (Q10.6) and dental caries (Q10.5).

Understanding of drug–microbiota interactions ranged from 42.9% to 52.6% (Figure 3, lower panel). The highest proportions were observed for drug metabolism involvement (Q11.3, 52.6%) and increased drug absorption (Q11.2, 49.7%), and lower proportions for reduced drug absorption (Q11.1, 42.9%) and prodrug activation (Q11.4, 43.7%). Differences by academic level were identified for increased drug absorption (Q11.2, *p* < 0.001), prodrug activation (Q11.4, *p* < 0.001), reduced drug absorption (Q11.1, *p* = 0.005), and drug accumulation within the microbiota (Q11.6, *p* = 0.041), with higher proportions among advanced students. No differences were observed for drug metabolism involvement (Q11.3) or toxic metabolite formation (Q11.5). When stratified by self-assessed knowledge, correct response rates increased across categories (Supplementary Figures, Figure S3). Differences were observed for drug metabolism involvement (Q11.3), toxic metabolite formation (Q11.5), prodrug activation (Q11.4), and reduced drug absorption (Q11.1), with correct responses remaining below 60% among students reporting Good or Very Good knowledge for most items.

### 3.3. Attitudes of Medical Students About Probiotics Training and Clinical Roles

**Figure 4:**
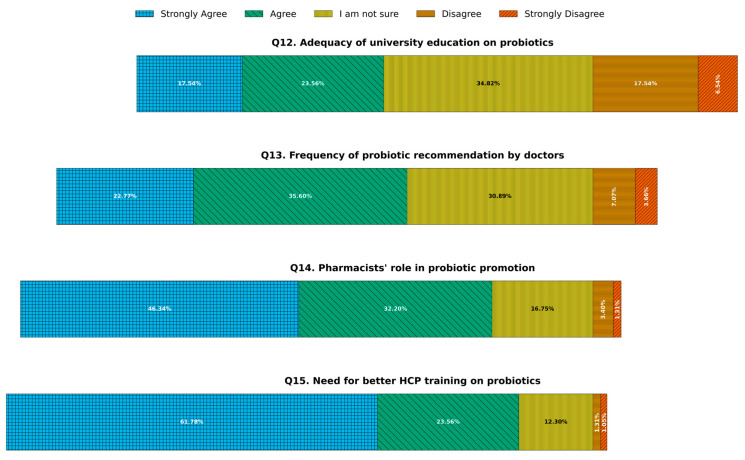
Likert scale results reflecting medical students’ perceptions on probiotics education and healthcare roles.

The overall median attitude score was 10 (IQR: 4.0) out of 16 points, corresponding to 62.5% of the maximum score. No differences were observed by sex, lifestyle habits, or chronic disease status. Differences were identified by prior instruction on microbiota and probiotics as well as by self-assessed knowledge (Table 3). In the multivariable linear regression analysis, prior instruction remained significantly associated with attitude scores, whereas self-assessed knowledge was not (Table 4). Compared with students reporting extensive prior lectures, those with limited or no instruction had lower scores. The model explained 4.3% of the variance in attitude scores (adjusted R^2^ = 0.038). Responses to individual items are shown in Figure 4. Agreement or strong agreement was reported for the role of pharmacists in promoting probiotic use (Q14, 78.5%) and for the need for improved training among healthcare professionals (Q15, 85.3%). For adequacy of university training (Q12), 41.1% agreed or strongly agreed, 34.8% were uncertain, and 24.1% disagreed. For physician recommendation of probiotics (Q13), 58.4% agreed or strongly agreed that physicians rarely recommend them, 30.9% were uncertain, and 10.7% disagreed. A positive correlation was observed between attitude and knowledge scores (r = 0.35, *p* < 0.001).

### 3.4. Probiotic Utilization Practices in Medical Students

**Figure 5:**
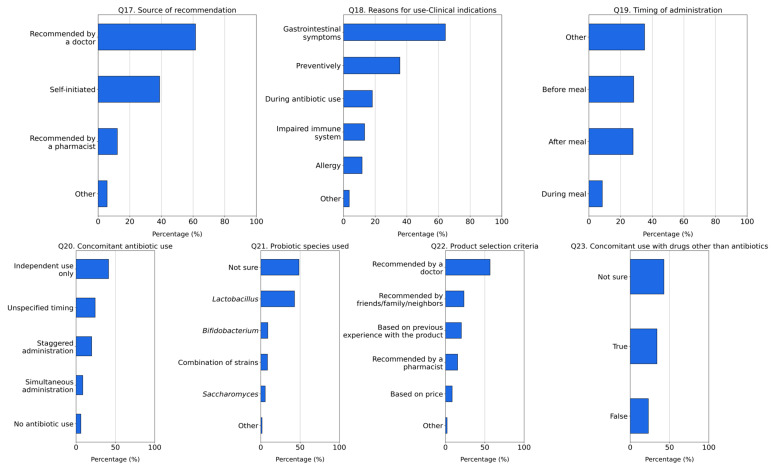
Probiotics usage practices among medical students covering source, indications, administration, and product choice.

Prior probiotic use was reported by 64.6% of students (18.8% once, 8.4% twice, 37.4% more than twice), while 35.3% had never used them. Among the 247 respondents reporting previous use, physician recommendation was reported by 61.5% (*n* = 152), self-initiated use by 38.9% (*n* = 96), pharmacist recommendation by 12.1% (*n* = 30), and other sources by 5.7% (Q17). Reported indications included gastrointestinal symptoms (64.4%, *n* = 159), preventive use (35.6%, *n* = 88), antibiotic-associated use (18.2%, *n* = 45), impaired immune function (13.4%, *n* = 33), allergies (11.7%, *n* = 29), and other reasons (3.6%, *n* = 9) (Q18). Regarding timing of administration (Q19), 35.2% (*n* = 87) reported not considering timing, 28.3% (*n* = 70) reported use before meals, 27.9% (*n* = 69) after meals, and 8.5% (*n* = 21) during meals (Figure 5, upper panels). For antibiotic co-administration (Q20), 41.3% reported independent use, 24.3% did not consider timing, 19.8% staggered administration, 8.5% used probiotics simultaneously, and 6.1% reported no antibiotic use. Knowledge of product composition (Q21) showed that 48.6% were unsure of the microorganisms contained. Reported genera included *Lactobacillus* (42.9%), *Bifidobacterium* (8.9%), combination formulations (8.5%), and *Saccharomyces* (5.7%). Factors influencing product selection (Q22) included physician recommendation (56.7%), advice from friends or family (23.5%), prior experience (20.2%), pharmacist recommendation (15.4%), and price (8.5%). Regarding concomitant use with non-antibiotic medications (Q23), 42.9% were unsure, 34.0% reported no use, and 23.1% reported use (Figure 5, lower panels).

## 4. Discussion

This study assessed knowledge, attitudes, and practices regarding gut microbiota and probiotics among medical students in Samborondón, Greater Guayaquil. Knowledge was uneven across conceptual, clinical, and pharmacological domains, with higher scores associated with academic progression and prior instruction, although gaps persisted even among advanced students. Self-perceived knowledge only partially aligned with objective performance, while reported practices revealed inconsistencies in administration, limited awareness of product composition, and uncertainty regarding drug interactions. Notably, beyond confirming overall limited knowledge, the study identifies critical gaps in safety-related knowledge and drug–microbiota interactions, alongside a mismatch between widespread probiotic use and limited understanding, highlighting challenges in translating microbiome science into clinical readiness.

Previous studies assessing knowledge of gut microbiota and probiotics among university students generally report moderate levels of understanding, with most participants demonstrating only intermediate knowledge despite favorable attitudes [27,28]. Similar patterns have been observed among medical, pharmacy, and dental students, where relatively few achieve high proficiency [29,30,31]. Consistent with these findings, the present study showed a 52.7% correct response rate, indicating limited knowledge, which may be overestimated due to self-selection bias. Academic progression and prior instruction were associated with higher scores, with final-year students scoring approximately six points higher than those in the basic cycle and those with extensive prior instruction scoring about five points higher. However, these factors explained only a modest proportion of the variance (~15%), suggesting that additional influences—such as individual learning behaviors, curricular differences, and external information sources—may contribute to knowledge variability [27,61].

Educational gradients similar to those observed in this study have been reported elsewhere, with senior students demonstrating higher knowledge scores, increased microbiota awareness during clinical training, and improved knowledge following formal coursework [28,29,32,33]. Despite these trends, important gaps persisted. Safety-related items, including probiotic use during pregnancy (23.3%) and childhood (23.6%), showed consistently low performance, even among advanced students. Similar deficiencies have been reported in health science students, particularly regarding probiotic indications and safety considerations [27,28]. Knowledge of drug–microbiota interactions was also limited, with correct response rates generally below 60%, consistent with previous findings indicating only partial awareness of these relationships [32,33]. These findings suggest that while academic training may improve foundational knowledge, clinically oriented aspects—particularly safety and pharmacological interactions—may be less consistently integrated within this cohort.

Previous studies among health science, dental, medical, and pharmacy students show that while basic probiotic concepts are generally recognized, knowledge of specific strains, clinical applications, and microbiota–drug interactions is more limited [27,29,31]. Probiotic benefits are also most commonly associated with gastrointestinal conditions, with lower awareness of other indications [27,31]. Similar patterns were observed in this study, with higher recognition of foundational concepts such as probiotics (59.4%) and synbiotics (57.6%), compared with lower accuracy in applied domains, including safety and pharmacological aspects. Recognition was highest for gastrointestinal conditions, particularly inflammatory bowel diseases (72.5%) and diarrhea (67.0%), whereas awareness of other applications, such as eczema prevention (34.3%), was lower. Knowledge of factors influencing microbiota composition was uneven: diet (86.4%) and antibiotic use (84.0%) were widely recognized, while mode of childbirth (42.4%) and orally administered medications (13.4%) were less frequently identified. Understanding of microbiota–drug interactions was also limited (42.9–52.6%), consistent with previous reports showing only partial awareness of these processes [29,32,33]. Overall, these findings indicate that while foundational concepts are relatively well retained, more complex and clinically integrated topics may be less consistently consolidated within this cohort.

Probiotic and microbiota-related knowledge is generally moderate across studies, with few students achieving high proficiency [27,28,29]. Similar patterns have been reported among healthcare professionals, where familiarity with probiotic mechanisms and indications remains incomplete [46], which suggests that self-assessed knowledge may only partially reflect actual understanding. In the present study, self-assessment showed partial concordance with objective performance: students reporting Good knowledge achieved higher scores than those reporting Poor or Very Poor familiarity. However, this relationship was not consistent, as those selecting Very Good did not significantly outperform other groups. Importantly, gaps persisted even among higher self-rated categories, particularly for safety-related items and drug–microbiota interactions. This misalignment indicates that perceived knowledge may overestimate actual competence, which could influence decisions regarding probiotic use, safety, and potential drug interactions.

Attitudes toward probiotics and microbiota-related education are generally favorable across students, healthcare professionals, and the general public, although negative perceptions may occur in groups with limited knowledge or misconceptions [27,28,31,34,35,36,37,38]. In this cohort, approximately two-thirds of participants expressed positive attitudes, with limited variability across demographic and academic subgroups. Prior formal instruction was associated with slightly higher attitude scores, whereas academic progression was not. Similar patterns have been reported elsewhere, where attitudes did not differ by general educational level but were higher among individuals with topic-specific training [39,40]. These findings show that attitudes remain relatively stable across training stages, with only modest influence from formal instruction. Students recognized the clinical relevance of probiotics, expressing strong agreement on the need for improved professional training and the role of pharmacists. However, fewer than half considered their university education adequate, indicating a perceived gap. Comparable results have been reported among Serbian students, where perceived training adequacy was also low despite generally positive attitudes [29]. A positive correlation between knowledge and attitudes was observed, consistent with previous studies showing that favorable perceptions can coexist with only moderate knowledge [28,35,36]. Together, these outcomes highlight the need for educational strategies that not only sustain positive attitudes but also strengthen the underlying scientific understanding.

Healthcare students frequently report active engagement with probiotics, often despite only moderate knowledge levels [27,41,42]. In this study, nearly two-thirds of participants reported prior use, with over one-third indicating repeated consumption, primarily for gastrointestinal symptoms and preventive purposes. However, understanding of microbiological composition and evidence-based indications remained limited. Similar patterns have been reported elsewhere, where regular use coexists with incomplete knowledge, suggesting that behavioral adoption may outpace scientific understanding [27,41]. For example, among Serbian healthcare students, nearly half were unable to identify the products they had consumed and many did not consider timing of administration [29]. Consistent with this, almost half of users in the present cohort could not identify the microorganisms in their products, and recognition beyond *Lactobacillus* was low, despite the strain-specific nature of probiotic effects [26,62]. Administration practices were also inconsistent. Over one-third of participants did not consider timing, despite evidence that intake during meals may improve bacterial survival [63,64]. Regarding antibiotic use, only a minority reported staggered or simultaneous administration, while many did not consider timing or used probiotics independently. Uncertainty extended to other medications, with a substantial proportion unsure about concomitant use. Similar findings have been described among medical and pharmacy students [29]. Given that probiotics may influence drug metabolism and reduce antibiotic-associated diarrhea when appropriately administered [25,65,66], these patterns suggest that common use is not consistently accompanied by informed practices, potentially limiting therapeutic benefit.

Multiple factors influence probiotic use, including knowledge, lifestyle, and access to guidance. Greater familiarity with probiotics is associated with higher likelihood of use, and individuals with healthier lifestyles may be more inclined to consume them [37,43]. Healthcare professionals also play a key role in shaping decisions [44]. In this study, physician recommendation was the main driver of both initiation and product selection, highlighting the influence of medical authority. In contrast, pharmacist involvement was limited, despite the relevance of probiotics to medication counseling. Similar variability has been reported elsewhere; while some studies identify pharmacists as important sources of guidance [29,46], others show that many individuals use probiotics without professional advice [45]. Informal sources also contributed to probiotic use in this cohort, including self-initiation, prior experience, and advice from family or peers, consistent with reports that media and social networks influence awareness and consumption [47,48]. These results suggest that probiotic use in this cohort may reflect a combination of professional guidance and informal influences, although the relative contribution of these factors cannot be determined.

Given the limited number of studies from Ecuador, comparison with findings from other countries provides useful context. Studies in Serbia, Turkey, Jordan, and Indonesia consistently report moderate knowledge levels among health science students, alongside gaps in clinically relevant domains such as safety in vulnerable populations, drug–microbiota interactions, and strain recognition [29,30,33,41]. Patterns of probiotic use are also similar, including frequent consumption, limited awareness of product composition, inconsistent administration practices, and reliance on physician recommendation [29]. Differences across settings are nonetheless evident. For example, Serbian students more frequently identified pharmacists as a source of guidance [29], whereas pharmacist involvement in the present cohort was limited. Such variation may reflect differences in healthcare system structure, professional roles, and the integration of microbiota-related content within curricula. It could be argued that while knowledge gaps appear consistent across settings, their practical expression may vary according to local educational and healthcare contexts.

This study has several limitations. First, the response rate was relatively modest (28.1%), and voluntary participation may have introduced selection bias, potentially overrepresenting students with prior interest in the topic. Second, the cross-sectional design limits interpretation to associations and precludes conclusions about causality or temporal changes. Third, the study was conducted in a single university setting and was intended as a setting-specific evaluation; therefore, the findings should not be generalized beyond similar academic environments. Future research should include multi-institutional and longitudinal designs, more representative sampling strategies, and additional analytical approaches to better explain variability in knowledge, attitudes, and practices.

Despite these limitations, this study provides a context-specific assessment of knowledge, attitudes, and practices among medical students in a major academic setting in Ecuador, where evidence remains limited. A key strength is the multi-domain approach, which captures both conceptual understanding and behavioral patterns. The results highlight areas that may benefit from improved curricular integration, particularly regarding safety in vulnerable populations, drug–microbiota interactions, clinical indications, and product literacy. These results may serve as a reference point for future studies in similar settings.

## 5. Conclusions

This study evaluated medical students’ knowledge, attitudes, and practices regarding gut microbiota and probiotics in a university setting in Greater Guayaquil, Ecuador. Knowledge levels were overall moderate but insufficient, with marked heterogeneity across domains. While students showed familiarity with general concepts, substantial gaps were observed in clinically relevant areas, including probiotic safety in vulnerable populations, non-gastrointestinal indications, and microbiota–drug interactions. Academic progression and prior instruction were associated with higher scores, although important deficiencies persisted across all levels. Attitudes toward probiotics and microbiota-related education were generally positive, and probiotic use was common. However, reported practices showed inconsistencies in administration timing, limited awareness of product composition, and uncertainty regarding concomitant drug use, indicating a mismatch between knowledge and application. These outcomes highlight areas that may benefit from improved curricular integration, particularly in clinically relevant and pharmacological domains, within similar educational settings.

## Figures and Tables

**Table 1:** Demographic characteristics and knowledge scores.

Variables	Number of Participants	%	Mean Score (SD) *	F-Values	*p*-Value	η^2^—Eta Squared	95% CI **	
*Sex*				2.92	0.08	0.002	−0.0026	0.02
Female	239	62.57	22.38 (9.2)					
Male	143	37.43	20.73 (9.0)					
*Level of Education*				12.77	<0.001	0.08	0.04	0.14
Basic (1st and 2nd years)	217	56.81	19.94 (9.2) ^a^					
Intermediate (3rd and 4th years)	115	30.10	23.20 (9.0) ^b^					
Advanced (5th and 6th years)	50	13.09	26.34 (6.8) ^b^					
*Lifestyle Habits*				1.01	0.400	−0.004	−0.002	0.04
Very good	35	9.16	21.34 (9.0)					
Good	107	28.01	22.48 (8.8)					
Fair	195	51.05	21.66 (9.3)					
Poor	30	7.85	22.47 (8.3)					
Very poor	15	3.93	17.6 (10.3)					
*Chronic diseases*				0.11	0.74	−0.003	−0.003	0.01
Yes	46	12.04	21.34 (9.0)					
No	336	87.96	21.82 (9.2)					
*Previous lectures on microbiota/probiotics*				15.85	<0.001	0.07	0.03	0.14
Yes (large extent)	77	20.16	26.71 (7.8) ^a^					
Yes (limited extent)	121	31.68	21.18 (8.8) ^b^					
No	184	48.17	20.07 (9.2) ^b^					
*Self-assessment of knowledge on microbiota/probiotics*				11.50	<0.001	0.10	0.05	0.18
Very good	15	3.93	22.73 (9.1) ^a,b^					
Good	71	18.59	24.93 (8.6) ^b^					
Fair	143	37.47	23.90 (8.5) ^b^					
Poor	116	30.37	18.73 (8.2) ^a^					
Very poor	37	9.69	16.54 (10.5) ^a^					

* Maximum knowledge score was 44/44. SD: Standard deviation. Different letters denote significant differences within demographics. ** Confidence intervals for η^2^.

**Table 2:** Results of the multiple linear regression on factors related with knowledge scores.

Variables	Coefficient	SE	t-Stat	*p*-Value	95% CI *	
*Level of Education*						
Advanced vs. Intermediate	−3.38	1.44	−2.35	0.019	−6.20	−0.55
Advanced vs. Basic	−5.14	1.35	−3.80	<0.001	−7.79	−2.48
*Previous lectures on microbiota/probiotics*						
Yes (large extent) vs. Yes (limited extent)	−3.39	1.33	−2.56	0.01	−6.00	−0.79
Yes (large extent) vs. No	−3.58	1.29	−2.77	0.006	−6.12	−1.03
*Self-assessment of knowledge on microbiota/probiotics*						
Good vs. Poor	−4.21	1.37	−3.07	0.002	−6.91	−1.51
Good vs. Very poor	−6.29	1.79	−3.51	<0.001	−9.81	−2.76

* Confidence interval. SE: Standard error; vs.: versus.

**Table 3:** Participant attitudinal scores.

Variables	Median Score (IQR) *	F-Values	*p*-Value	η^2^—Eta Squared	95% CI **	
*Sex*		0.53	0.46	−0.001	−0.003	0.02
Female	10.00 (4.0)					
Male	10.00 (4.0)					
*Level of Education*		2.50	0.29	0.001	−0.005	0.03
Basic (1st and 2nd years)	10.00 (3.0)					
Intermediate (3rd and 4th years)	10.00 (4.0)					
Advanced (5th and 6th years)	10.00 (3.8)					
*Lifestyle Habits*		6.47	0.16	0.007	−0.005	0.05
Very good	10.00 (3.0)					
Good	10.00 (3.0)					
Fair	10.00 (3.0)					
Poor	11.00 (3.0)					
Very poor	9.00 (3.5)					
*Chronic diseases*		0.07	0.79	−0.002	−0.0026	0.01
Yes	10.00 (4.0)					
No	10.00 (4.0)					
*Previous lectures on microbiota/probiotics*		15.82	<0.001	0.04	0.009	0.09
Yes (large extent)	11.00 (2.0) ^a^					
Yes (limited extent)	9.00 (4.0) ^b^					
No	10.00 (3.3) ^b^					
*Self-assessment of knowledge on microbiota/probiotics*		19.91	<0.001	0.04	0.01	0.1
Very good	11.00 (2.0) ^a^					
Good	11.00 (3.0) ^a^					
Fair	10.00 (3.0) ^b^					
Poor	9.00 (4.0) ^b,c^					
Very poor	8.00 (5.0) ^c^					

* Maximum attitudinal score was 16/16. IQR: interquartile ranges. Different letters denote significant differences within demographics. ** Confidence intervals for η^2^.

**Table 4:** Results of the multiple linear regression on factors related with attitudinal scores.

Variables	Coefficient	SE	t-Stat	*p*-Value	95% CI *	
*Previous lectures on microbiota/probiotics*						
Yes (large extent) vs. Yes (limited extent)	−1.59	0.48	−3.33	<0.001	−2.53	−0.65
Yes (large extent) vs. No	−1.80	0.45	−4.04	<0.001	−2.68	−0.93

* Confidence interval. SE: Standard error; vs.: versus.

## Data Availability

The original contributions presented in this study are included in the article/Supplementary Materials.

## Supplementary Materials

The following supporting information can be downloaded at: https://www.mdpi.com/article/10.3390/healthcare14111551/s1, Supplementary Materials, Survey; Supplementary Results, Raw data; Supplementary Figures, Figure S1: Students’ Correct Response Rate on Basic and Applied Probiotics Questions; Supplementary Figures, Figure S2: Knowledge Assessment on Microbiota and Probiotics Knowledge, including Factors Affecting Gut microbiota Composition (Upper Panel) and Microorganisms Recognized as Probiotics (Lower Panel); Supplementary Figures, Figure S3: Accuracy on Statements Regarding Probiotics in Clinical Practice with the Upper Panel Showing Therapeutic Efficacy and the Lower Panel Showing Pharmacological Interactions.

## References

[1] Lynch S.V., Pedersen O. (2016). The human intestinal microbiome in health and disease. N. Engl. J. Med..

[2] Thursby E., Juge N. (2017). Introduction to the human gut microbiota. Biochem. J..

[3] Fassarella M., Blaak E.E., Penders J., Nauta A., Smidt H., Zoetendal E.G. (2021). Gut microbiome stability and resilience: Elucidating the response to perturbations in order to modulate gut health. Gut.

[4] Lloyd-Price J., Abu-Ali G., Huttenhower C. (2016). The healthy human microbiome. Genome Med..

[5] Schnabl B., Brenner D.A. (2014). Interactions between the intestinal microbiome and liver diseases. Gastroenterology.

[6] Fan Y., Pedersen O. (2021). Gut microbiota in human metabolic health and disease. Nat. Rev. Microbiol..

[7] Wong S.H., Yu J. (2019). Gut microbiota in colorectal cancer: Mechanisms of action and clinical applications. Nat. Rev. Gastroenterol. Hepatol..

[8] Suez J., Zmora N., Segal E., Elinav E. (2019). The pros, cons, and many unknowns of probiotics. Nat. Med..

[9] FAO/WHO (2001). Health and Nutritional Properties of Probiotics in Food Including Powder Milk with Live Lactic Acid Bacteria. http://www.fao.org/3/a-a0512e.pdf.

[10] Hill C., Guarner F., Reid G., Gibson G.R., Merenstein D.J., Pot B., Morelli L., Canani R.B., Flint H.J., Salminen S. (2014). Expert consensus document: The international scientific association for probiotics and prebiotics consensus statement on the scope and appropriate use of the term probiotic. Nat. Rev. Gastroenterol. Hepatol..

[11] Fijan S. (2014). Microorganisms with claimed probiotic properties: An overview of recent literature. Int. J. Environ. Res. Public Health.

[12] Markowiak P., Śliżewska K. (2017). Effects of probiotics, prebiotics, and synbiotics on human health. Nutrients.

[13] Markowiak P., Śliżewska K. (2018). The role of probiotics, prebiotics and synbiotics in animal nutrition. Gut Pathog..

[14] Alp D., Kuleaşan H. (2019). Adhesion mechanisms of lactic acid bacteria: Conventional and novel approaches for testing. World J. Microbiol. Biotechnol..

[15] Zheng Y., Zhang Z., Tang P., Wu Y., Zhang A., Li D., Wang C., Wan X., Zhang Q., Liu T. (2023). Probiotics fortify intestinal barrier function: A systematic review and meta-analysis of randomized trials. Front. Immunol..

[16] Sorbara M.T., Pamer E.G. (2019). Interbacterial mechanisms of colonization resistance and the strategies pathogens use to overcome them. Mucosal Immunol..

[17] Wang J., Ji H., Wang S., Liu H., Zhang W., Zhang D., Wang Y. (2018). Probiotic *Lactobacillus plantarum* promotes intestinal barrier function by strengthening the epithelium and modulating gut microbiota. Front. Microbiol..

[18] Arena M.P., Capozzi V., Russo P., Drider D., Spano G., Fiocco D. (2018). Immunobiosis and probiosis: Antimicrobial activity of lactic acid bacteria with a focus on their antiviral and antifungal properties. Appl. Microbiol. Biotechnol..

[19] Ashraf R., Shah N.P. (2014). Immune system stimulation by probiotic microorganisms. Crit. Rev. Food Sci. Nutr..

[20] Li Q., Zheng T., Ding H., Chen J., Li B., Zhang Q., Ma S., Zhang J., Wang X., Liu Y. (2023). Exploring the benefits of probiotics in gut inflammation and diarrhea—From an antioxidant perspective. Antioxidants.

[21] Vallejos O.P., Bueno S.M., Kalergis A.M. (2025). Probiotics in inflammatory bowel disease: Microbial modulation and therapeutic prospects. Trends Mol. Med..

[22] Cuello-Garcia C.A., Brożek J.L., Fiocchi A., Pawankar R., Yepes-Nuñez J.J., Terracciano L., Gandhi S., Agarwal A., Zhang Y., Schünemann H.J. (2015). Probiotics for the prevention of allergy: A systematic review and meta-analysis of randomized controlled trials. J. Allergy Clin. Immunol..

[23] Mei Z., Li D. (2022). The role of probiotics in vaginal health. Front. Cell. Infect. Microbiol..

[24] Zimmermann M., Zimmermann-Kogadeeva M., Wegmann R., Goodman A.L. (2019). Mapping human microbiome drug metabolism by gut bacteria and their genes. Nature.

[25] Purdel C., Ungurianu A., Adam-Dima I., Margină D. (2023). Exploring the potential impact of probiotic use on drug metabolism and efficacy. Biomed. Pharmacother..

[26] McFarland L.V., Evans C.T., Goldstein E.J. (2018). Strain-specificity and disease-specificity of probiotic efficacy: A systematic review and meta-analysis. Front. Med..

[27] Al hossan A.A., Syed W., Babelghaith S.D., Al Arifi M.N. (2024). Knowledge, attitude, and practice of probiotics among Saudi health care students—A cross-sectional study from Saudi university in Riyadh Saudi Arabia. INQUIRY J. Health Care Organ. Provis. Financ..

[28] Webb M., Singh K.H. (2026). Knowledge, attitude and perception toward probiotics among university students. Nutr. Food Sci..

[29] Đanić M., Marković N., Ostojić T., Kojić M., Lazarević S., Mikov M., Pavlović N. (2024). Intestinal microbiota, probiotics and their interactions with drugs: Knowledge, attitudes and practices of health science students in Serbia. BMC Med. Educ..

[30] Davarci İ., Davarci P.Z. (2025). Microbiota awareness levels of medical students: The case of Trakya university. BMC Med. Educ..

[31] Babina K., Salikhova D., Polyakova M., Zaytsev A., Egiazaryan A., Novozhilova N. (2023). Knowledge and attitude towards probiotics among dental students and teachers: A cross-sectional survey. Dent. J..

[32] Helisz P., Dziubanek G., Krupa-Kotara K., Gwioździk W., Grajek M., Głogowska-Ligus J. (2022). Colorectal cancer and the role of the gut microbiota—Do medical students know more than other young people?—Cross-sectional study. Nutrients.

[33] Abu-Humaidan A.H.A., Alrawabdeh J.A., Theeb L.S., Hamadneh Y.I., Omari M.B. (2021). Evaluating knowledge of human microbiota among university students in Jordan, an online cross-sectional survey. Int. J. Environ. Res. Public Health.

[34] Soni R., Tank K., Jain N. (2018). Knowledge, attitude and practice of health professionals about probiotic use in Ahmedabad, India. Nutr. Food Sci..

[35] Wilson Z., Whitehead K. (2019). A cross sectional survey to assess healthcare professionals’ attitudes to and understanding of probiotics. Clin. Nutr. ESPEN.

[36] Ayyash M., Al-Najjar M.A., Jaber K., Ayyash L., Abu-Farha R. (2021). Assessment of public knowledge and perception about the use of probiotics. Eur. J. Integr. Med..

[37] Khalesi S., Vandelanotte C., Thwaite T., Russell A.M., Dawson D., Williams S.L. (2021). Awareness and attitudes of gut health, probiotics and prebiotics in Australian adults. J. Diet. Suppl..

[38] Wang J., Jia Q., Yang C., Gao Y., Yang J., Wang L., Li L., Tie Y., Feng Z. (2025). Knowledge, attitude and practice toward probiotics among the general population: A cross-sectional study. Sci. Rep..

[39] Đanić M., Pavlović N., Ostojić T., Marković N., Stanimirov B., Lazarević S., Mikov M. (2025). Parental knowledge, attitudes, and practices on probiotic use in preschool children in Serbia: A cross-sectional study. Front. Immunol..

[40] Caiminagua D.B.B., Villegas J.L., Larrea-Álvarez C.M., Pincay N.M.M., Šefcová M.A., Larrea-Álvarez M. (2026). Genetic knowledge and attitudes toward genomics across academic disciplines, a cross-sectional survey of university students in Samborondón, greater Guayaquil, Ecuador. J. Community Genet..

[41] Rahmah P.A., Khairani A.F., Atik N., Arisanti N., Fatimah S.N. (2021). Correlation of knowledge, attitude, and practice toward probiotics for the digestive system among health science students. J. Multidiscip. Healthc..

[42] Alqaydi T.K., Bedir A.S., Abu-Elsaoud A.M., El-Tarabily K.A., Al Raish S.M. (2024). An Assessment of the knowledge, attitude, and practice of probiotics and prebiotics among the population of the United Arab Emirates. Foods.

[43] Cemiloğlu S., Yılmaz H.Ö. (2025). The association between probiotic food consumption, microbiota awareness and gastrointestinal symptoms among healthcare professionals: A cross-sectional study. Discov. Public Health.

[44] Schultz M., Baranchi A., Thurston L., Yu Y.C., Wang L., Chen J., Sapsford M., Chung J., Binsadiq M., Craig L. (2011). Consumer demographics and expectations of probiotic therapy in New Zealand: Results of a large telephone survey. N. Z. Med. J..

[45] Draper K., Ley C., Parsonnet J. (2017). A survey of probiotic use practices among patients at a tertiary medical centre. Benef. Microbes.

[46] Başar Güneş H., Bayraktar Ekincioğlu A., Karakan T., Demirkan K. (2024). Assessment of knowledge and attitudes of physicians and pharmacists on probiotics: A cross-sectional survey. Turk. J. Pharm. Sci..

[47] Chin-Lee B., Curry W.J., Fetterman J., Graybill M.A., Karpa K. (2014). Patient experience and use of probiotics in community-based health care settings. Patient Prefer. Adherence.

[48] Guner U.C., Kissal A. (2021). Mothers’ knowledge, attitudes and practices regarding probiotic use during pregnancy and for their infants in Turkey. Public Health Nutr..

[49] Carrillo J., Delgado B., Kosik R.O., Huang L., Zhao X., Su T.P., Wang S.J., Chen Q., Fan A.P.C. (2013). Medical education in Ecuador. Med. Teach..

[50] Mora Pincay N.M., Villegas J.L., Larrea-Álvarez C.M., Briones Caiminagua D.B., Torres-Elizalde L., Šefcová M.A., Larrea-Álvarez M. (2025). A cross-sectional study assessing antibiotic resistance awareness among university students in Samborondón, Greater Guayaquil, Ecuador. Antibiotics.

[51] Ortega-Paredes D., Larrea-Álvarez C.M., Torres-Elizalde L., de Janon S., Vinueza-Burgos C., Hidalgo-Arellano L., Šefcová M.A., Molina-Cuasapaz G., Fernandez-Moreira E., Larrea-Álvarez M. (2022). Antibiotic resistance awareness among undergraduate students in Quito, Ecuador. Antibiotics.

[52] Jaramillo-Aguilar D.S., Simbaña-Rivera K. (2024). Genetic knowledge and attitudes towards genetic testing among final-year medical students at a public university in Ecuador. Front. Med..

[53] Ortega-Paredes D., Larrea-Álvarez C.M., Jijón S.I., Loaiza K., Šefcová M.A., Molina-Cuasapaz G., Barba P., Vinueza-Burgos C., Fernandez-Moreira E., Ramírez H. (2021). A cross-sectional study to assess knowledge of COVID-19 among undergraduate students in North-Central Ecuador. Int. J. Environ. Res. Public Health.

[54] Argote S., Bucheli D., Páez A., Rovayo C., Freire D., Pila P., Sánchez J.D. (2025). The knowledge-attitude paradox in analgesic self-medication among university students in Ecuador: A cross-sectional study and predictive modeling analysis. Public Health Chall..

[55] Vásquez-Barberán S.L.Á., Mesache-Villagómez M.A., Arroyo-Lalama E.M., Vaca-Altamirano G.L. (2023). Conocimiento sobre el uso de probióticos para la prevención de caries dentales. Rev. Cienc. Médicas Pinar Río.

[56] (2022). Instituto Nacional de Estadística y Censos (INEC) Proyecciones Poblacionales. https://www.ecuadorencifras.gob.ec/proyecciones-poblacionales/.

[57] Anderson L.W., Krathwohl D.R. (2001). A Taxonomy for Learning, Teaching, and Assessing: A Revision of Bloom’s Taxonomy of Educational Objectives.

[58] Raosoft Inc. (2004). Sample Size Calculator. https://raosoftcalculator.com.

[59] Lee D.K. (2016). Alternatives to P value: Confidence interval and effect size. Korean J. Anesthesiol..

[60] Halsey L.G. (2019). The reign of the p-value is over: What alternative analyses could we employ to fill the power vacuum?. Biol. Lett..

[61] Aldabayan Y.S., Bano R., Huwaikem M., Al-Hashim S. (2025). Knowledge attitudes and practices regarding the use of prebiotics and probiotics for respiratory infections among Saudi healthcare students. Sci. Rep..

[62] Maslennikov R., Gosteeva E., Ananeva V., Korshunova L., Kravtsowa A., Poluektova E., Ulyanin A., Sigidaev A., Kikhasurova P., Ivashkin V. (2026). Strain-specific systematic review with meta-analysis of probiotics efficacy in the treatment of irritable bowel syndrome. J. Clin. Med..

[63] Wang J., Wu P., Chen X.D., Yu A., Dhital S. (2025). Effect of food matrix and administration timing on the survival of *Lactobacillus rhamnosus* GG during in vitro gastrointestinal digestion. Foods.

[64] Tompkins T.A., Mainville I., Arcand Y. (2011). The impact of meals on a probiotic during transit through a model of the human upper gastrointestinal tract. Benef. Microbes.

[65] Wanyama H., Akhtar T.S., Abbas S. (2025). Probiotic use reduces the incidence of antibiotic-associated diarrhea among adult patients: A meta-analysis. Prz. Gastroenterol..

[66] Hempel S., Newberry S.J., Maher A.R., Wang Z., Miles J.N., Shanman R., Johnsen B., Shekelle P.G. (2012). Probiotics for the prevention and treatment of antibiotic-associated diarrhea: A systematic review and meta-analysis. JAMA.

