# Supplementary material for: Knowledge, Attitudes, and Practices Regarding Gut Microbiota and Probiotics Among Ecuadorian Medical Students
Source: Healthcare (Basel). 2026 Jun 2;14(11):1551. doi: 10.3390/healthcare14111551 (PMC13256382; doi:10.3390/healthcare14111551)
Supplement: Supplementary file 1 [file healthcare-14-01551-s001.zip › Supplemetary Figures.pdf]

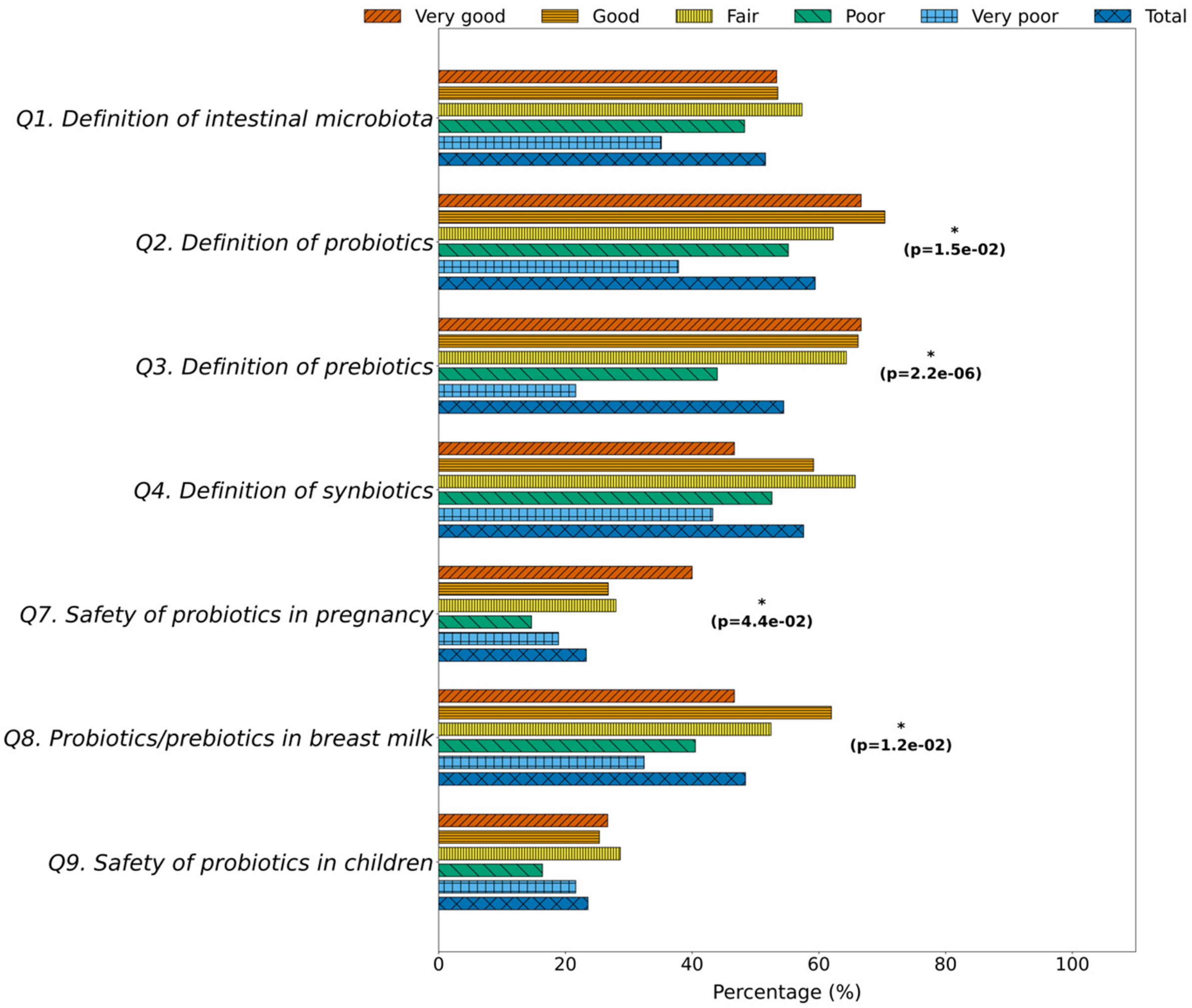

Figure S1. Students' Correct Response Rate on Basic and Applied Probiotics Questions. * indicates significant differences between levels of self-assessed knowledge.

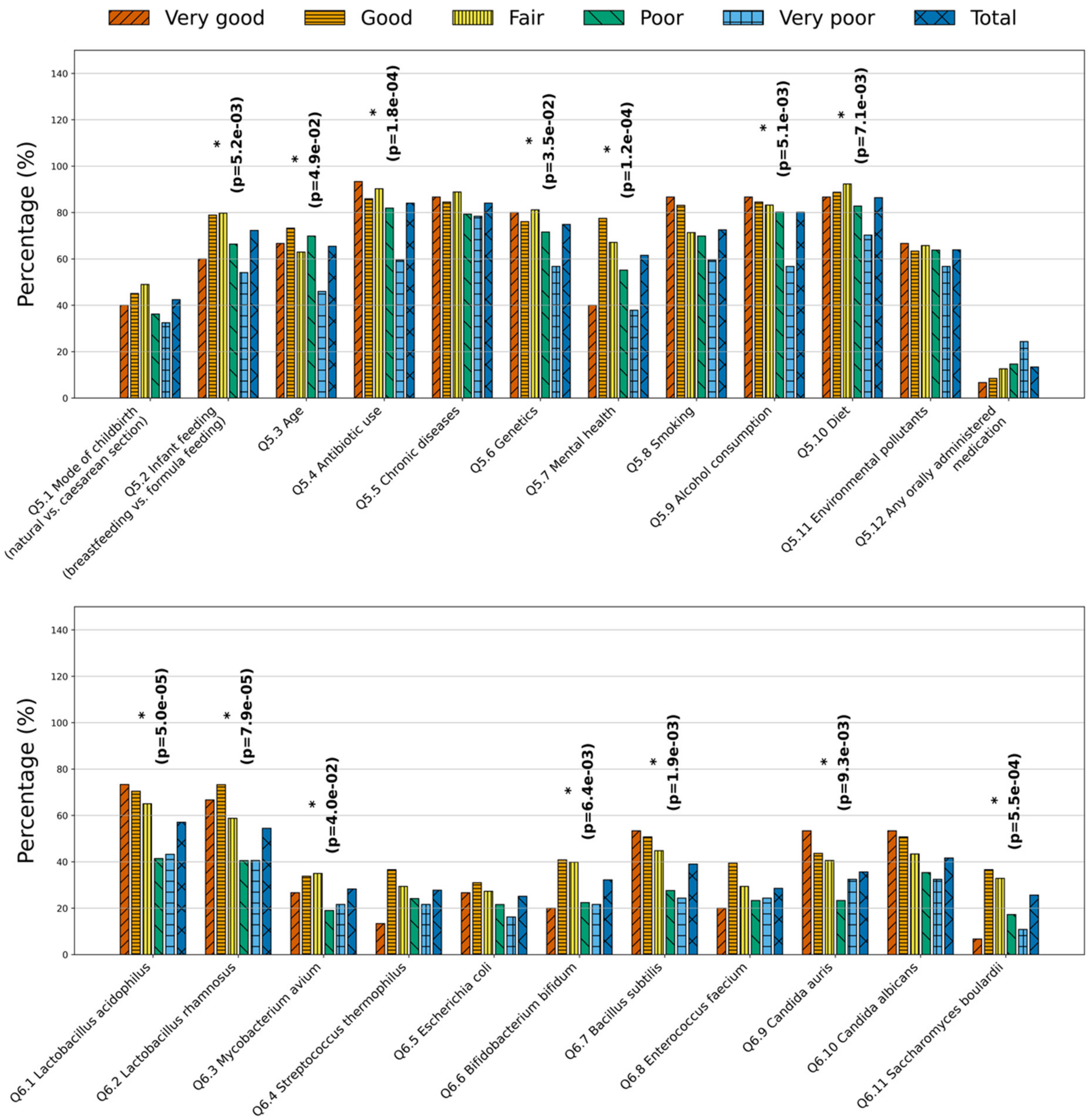

Figure S2. Knowledge Assessment on Microbiota and Probiotics Knowledge, including Factors Affecting Gut Microbiota Composition (Upper Panel) and Microorganisms Recognized as Probiotics (Lower Panel). * indicates significant differences between levels of self-assessed knowledge.

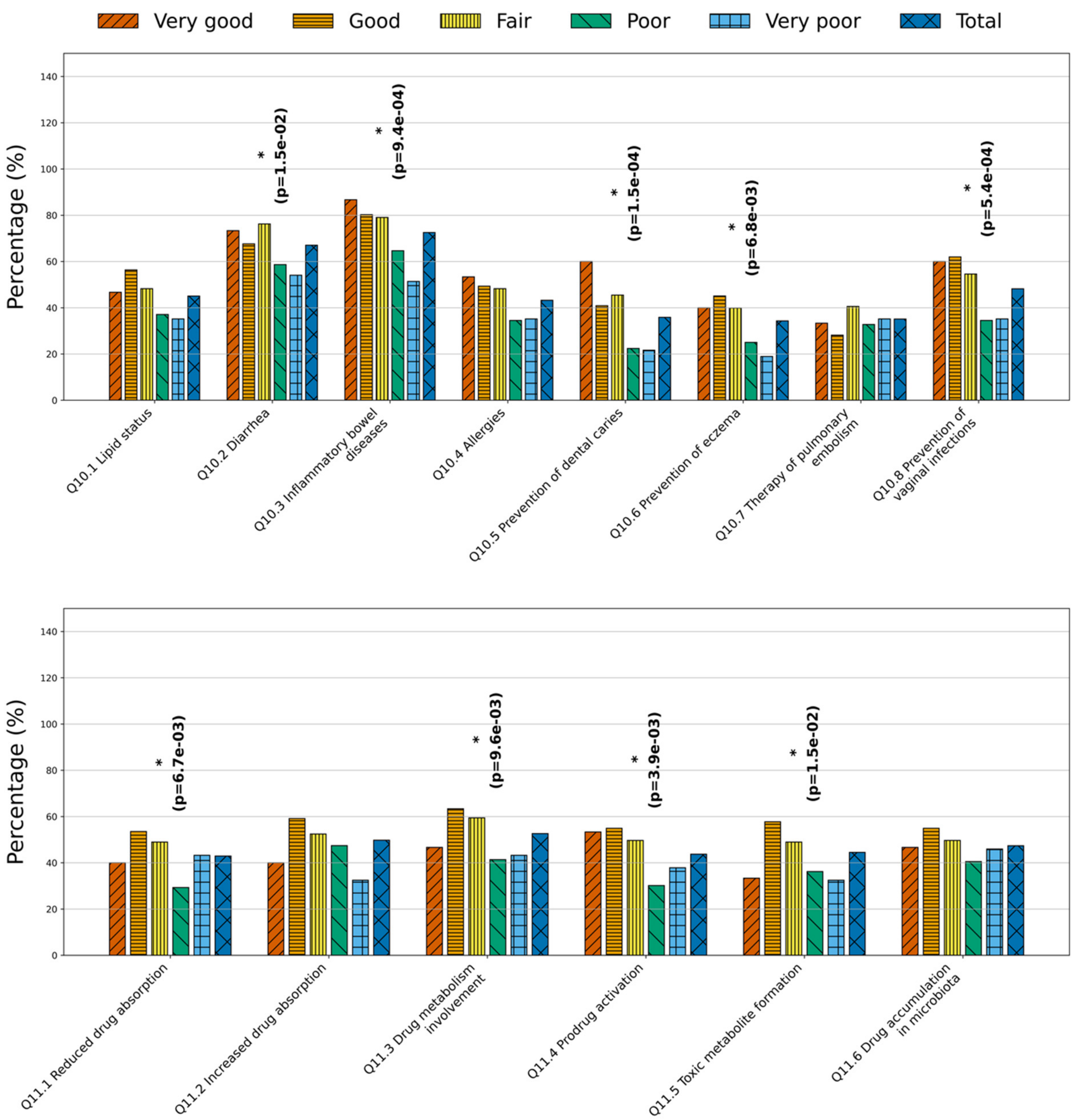

Figure S3. Accuracy on Statements Regarding Probiotics in Clinical Practice with the Upper Panel Showing Therapeutic Efficacy and the Lower Panel Showing Pharmacological Interactions. * indicates significant differences between levels of self-assessed knowledge.
