# Supplementary material for: Knowledge, Attitudes, and Practices Regarding Gut Microbiota and Probiotics Among Ecuadorian Medical Students
Source: Healthcare (Basel). 2026 Jun 2;14(11):1551. doi: 10.3390/healthcare14111551 (PMC13256382; doi:10.3390/healthcare14111551)
Supplement: Supplementary file 1 [file healthcare-14-01551-s001.zip › Supplementary Materials, Survey.pdf]

PROBIOTICS_ INTESTINAL MICROBIOTA questionnaire

Section 1: General information

i. *What is your gender?*
- ☐ Male
- ☐ Female
- ☐ Other

ii. *Study year:*
- ☐ First
- ☐ Second
- ☐ Third
- ☐ Fourth
- ☐ Fifth
- ☐ Sixth
- ☐ Other:

iii. *How would you rate your lifestyle and dietary habits?*

- ☐ Very good
- ☐ Good
- ☐ Fair
- ☐ Poor
- ☐ Very poor

iv. *Do you suffer from any chronic diseases?*[#]

- ☐ Yes
- ☐ No

[#] Chronic diseases are conditions that last one year or more and require ongoing medical attention (e.g. hypertension, diabetes, asthma, thyroid diseases, epilepsy...)

v. *Have you had any lectures related to gut microbiota and probiotics during your studies so far?*

- ☐ Yes, to a large extent
- ☐ Yes, to a small or insignificant extent
- ☐ Not at all

If yes, please specify the course: ____

vi. *Where do you get information about probiotics? (check more than one if applicable)*

- ☐ On the Internet
- ☐ From friends/family/neighbors
- ☐ From doctors
- ☐ From pharmacists (at the pharmacy)
- ☐ At university
- ☐ From scientific papers/journals
- ☐ Other: _______

vii. *How would you rate your knowledge about intestinal microbiota and probiotics?*

- ☐ Very good
- ☐ Good
- ☐ Fair
- ☐ Poor
- ☐ Very poor

Section 2: Knowledge

(represents the correct answers)*

Q1. What is intestinal microbiota?

- **All microorganisms that colonize the gastrointestinal tract***
- All bacterial species that colonize the gastrointestinal tract
- All metabolic products of bacteria in the gastrointestinal tract that exert positive effects on human health
- Pathogenic bacteria that are normally present in the gastrointestinal tract
- Synonym for probiotics

Q2. What are probiotics?

- Any microorganism administered in the form of food or supplements
- Any microorganism capable of adhering to the intestinal mucosa
- **Live microorganisms that exert positive effects on the host's health when administered in adequate amounts***
- Dead microorganisms that exert positive effects on the host's health when administered in adequate amounts
- Metabolic products of bacteria that exert positive effects on the host's health when administered in adequate amounts

Q3. What are prebiotics?

- Synonym for probiotics
- Live microorganisms that exert positive effects on the host's health when administered in adequate amounts
- Dead microorganisms that exert positive effects on the host's health when administered in adequate amounts
- Metabolically active products of the intestinal microbiota
- **Non-digestible food ingredients that selectively stimulate the growth or activity of the intestinal microbiota***

Q4. What are synbiotics?

- Synonym for probiotics
- Synonym for prebiotics
- **Combination of probiotics and prebiotics***
- Metabolically active products of the intestinal microbiota
- Non-digestible food ingredients that selectively stimulate the growth or activity of the intestinal microbiota

Q5. Which of the following factors may affect the composition of the intestinal microbiota?

	Read each statement and check a box (True, False, or I don't know) according to your knowledge.	True	False	I don't know
Q5.1.	**Mode of childbirth (natural vs. caesarean section)***			
Q5.2.	**Infant feeding (breastfeeding vs. formula feeding)***			
Q5.3.	**Age***			
Q5.4.	**Antibiotic use***			
Q5.5.	**Chronic diseases***			
Q5.6.	**Genetics***			
Q5.7.	**Mental health***			
Q5.8.	**Smoking***			
Q5.9.	**Alcohol consumption***			
Q5.10.	**Diet***			
Q5.11.	**Environmental pollutants***			
Q5.12.	Any orally administered medication			

Q6. Which of the following species can be used as probiotics?

	Read each statement and check a box (True, False, or I don't know) according to your knowledge.	True	False	I don’t know
Q6.1.	***Lactobacillus acidophilus******			
Q6.2.	***Lactobacillus rhamnosus******			
Q6.3.	*Mycobacterium avium*			
Q6.4.	***Streptococcus thermophilus******			
Q6.5.	***Escherichia coli******			
Q6.6.	***Bifidobacterium bifidum******			
Q6.7.	***Bacillus subtilis******			
Q6.8.	***Enterococcus faecium******			
Q6.9.	*Candida auris*			
Q6.10.	*Candida albicans*			
Q6.11.	***Saccharomyces boulardii******			

Q7. In pregnant women, probiotics are generally considered safe:

- **Throughout the entire pregnancy***
- In the second and third trimester
- Only in the third trimester
- Their use during pregnancy is not recommended
- I am not sure

Q8. Breast milk contains:

- Only probiotics
- Only prebiotics
- **Both probiotics and prebiotics***
- Neither probiotics nor prebiotics
- I am not sure

Q9. In children, probiotics are generally considered safe:

- **From birth***
- From the sixth month of life
- From the second year of life
- I am not sure

Q10. Which of the following statements regarding probiotics efficacy in certain indications is/are true?

	Read each statement and check a box (True, False, or I don't know) according to your knowledge.	True	False	I don’t know
Q10.1.	**Probiotics have a positive effect on lipid status***			
Q10.2.	**Probiotics are effective in diarrhea treatment***			
Q10.3.	**Probiotics are effective in treatment of inflammatory bowel diseases***			
Q10.4.	**Probiotics can be beneficial in allergic conditions***			
Q10.5.	**Probiotics can be beneficial in the prevention of dental caries***			
Q10.6.	**Probiotics can be beneficial in the prevention of eczema***			
Q10.7.	Probiotics are effective in the therapy of pulmonary embolism			
Q10.8.	**Probiotics can be beneficial in the prevention of vaginal infections***			

Q11. Which of the following statements regarding the interactions between probiotics and intestinal microbiota with drugs is/are true?

	Read each statement and check a box (True, False, or I don't know) according to your knowledge.	True	False	I don’t know
Q11.1.	**Probiotics can decrease the absorption of certain drugs***			
Q11.2.	**Probiotics can increase the absorption of certain drugs***			
Q11.3.	**Probiotics are involved in the metabolism of certain drugs***			
Q11.4.	**Intestinal microbiota can lead to the activation of certain prodrugs***			
Q11.5.	**Intestinal microbiota can lead to the formation of toxic metabolites of certain drugs***			
Q11.6.	**Certain drugs can accumulate in the gut microbiota and probiotics***			

Section 3: ATTITUDES

**Please indicate the degree to which you agree with the following statements:**	Strongly agree	Agree	I'm not sure	Disagree	Strongly disagree
Q12. I believe that I get enough information about the benefits of probiotics at the university					
Q13. I believe that doctors rarely recommend the use of probiotics					
Q14. I believe that pharmacists should play a significant role in promoting the use of probiotics					
Q15. I believe that healthcare professionals should be better informed about probiotics					

Section 4: EXPERIENCE AND BEHAVIOUR

Q16. So far, I have used probiotics:

- Once
- Two times
- More than two times
- Never

If you answered the previous question affirmatively, please answer the following questions (17-23). If you answered that you have never used them, we thank you for completing the questionnaire.

Q17. My decision to use probiotics was: (check more than one if applicable)

- Based on a doctor's recommendation
- Based on a pharmacist's recommendation at the pharmacy
- Self-initiated
- Other: ______

Q18. The indications for which I used probiotics were: (check more than one if applicable)

- Gastrointestinal symptoms
- Impaired immune system
- Allergy
- Preventively (to improve general well-being)
- During antibiotic use
- Other: _____

Q19. I was taking probiotics:

- Before meal
- After meal
- During meal
- I did not pay attention

Q20. While I was taking antibiotics:

- I took probiotics at a different time from the antibiotic
- I took probiotics at the same time as the antibiotic
- I didn't pay attention to the time of administration
- I haven't taken probiotics with antibiotics, only independently
- I have never taken antibiotics

Q21. Which bacteria were present in probiotic products you used? (check more than one if applicable)

- *Lactobacillus*
- *Bifidobacterium*
- *Saccharomyces*
- Combination of strains
- I am not sure
- Other: _____

Q22. How did you choose the specific probiotic product? (check more than one if applicable)

- Based on the doctor's recommendation
- Based on the pharmacist's recommendation
- Based on the recommendation of friends/family/neighbors
- Based on previous positive experience with the same product
- Based on the price
- Other: ______

Q23. I didn't use probiotics while taking other medications (other than antibiotics).

- True
- False
- I am not sure
